# Integrative Bioinformatics Analysis Reveals a Transcription Factor EB-Driven MicroRNA Regulatory Network in Endothelial Cells

**DOI:** 10.3390/ijms25137123

**Published:** 2024-06-28

**Authors:** Teresa Gravina, Francesco Favero, Stefania Rosano, Sushant Parab, Alejandra Diaz Alcalde, Federico Bussolino, Gabriella Doronzo, Davide Corà

**Affiliations:** 1 Department of Translational Medicine, University of Piemonte Orientale, 28100 Novara, Italy; teresa.gravina@uniupo.it (T.G.); francesco.favero@uniupo.it (F.F.); 2 Center for Translational Research on Allergic and Autoimmune Diseases (CAAD), University of Piemonte Orientale, 28100 Novara, Italy; 3 Department of Oncology, University of Torino, 10124 Orbassano, Italy; stefy.rosano@gmail.com (S.R.); sushant.parab@ircc.it (S.P.); alejandra.diazalcalde@ircc.it (A.D.A.); gabriella.doronzo@unito.it (G.D.); 4 Candiolo Cancer Institute, IRCCS-FPO, 10060 Candiolo, Italy

**Keywords:** transcription factor networks, TFEB, microRNAs

## Abstract

Various human diseases are triggered by molecular alterations influencing the fine-tuned expression and activity of transcription factors, usually due to imbalances in targets including protein-coding genes and non-coding RNAs, such as microRNAs (miRNAs). The transcription factor EB (TFEB) modulates human cellular networks, overseeing lysosomal biogenesis and function, plasma–membrane trafficking, autophagic flux, and cell cycle progression. In endothelial cells (ECs), TFEB is essential for the maintenance of endothelial integrity and function, ensuring vascular health. However, the comprehensive regulatory network orchestrated by TFEB remains poorly understood. Here, we provide novel mechanistic insights into how TFEB regulates the transcriptional landscape in primary human umbilical vein ECs (HUVECs), using an integrated approach combining high-throughput experimental data with dedicated bioinformatics analysis. By analyzing HUVECs ectopically expressing TFEB using ChIP-seq and examining both polyadenylated mRNA and small RNA sequencing data from TFEB-silenced HUVECs, we have developed a bioinformatics pipeline mapping the different gene regulatory interactions driven by TFEB. We show that TFEB directly regulates multiple miRNAs, which in turn post-transcriptionally modulate a broad network of target genes, significantly expanding the repertoire of gene programs influenced by this transcription factor. These insights may have significant implications for vascular biology and the development of novel therapeutics for vascular disease.

## 1. Introduction

Transcription factor EB (TFEB) belongs to the microphthalmia/transcription factor E (MiT/TFE) family, characterized by its basic helix–loop–helix (bHLH) and leucine–zipper (Zip) domains, which comprise four bHLH-Zip transcription factors: the microphthalmia-associated transcription factor (MITF), TFEB, transcription factor EC (TFEC), and transcription factor E3 (TFE3), all of which are evolutionarily conserved [1,2,3].

TFEB functions through the formation of homodimers or heterodimers with other MiT/TFE family members, enabling the binding of DNA to “coordinated lysosomal expression and regulation (CLEAR)” elements in the promoter regions of the target genes, ultimately leading to the activation of gene transcription [4,5,6]. The network of genes containing CLEAR elements in their promoter region is primarily associated with lysosomal catabolic activity, autophagy, exo- and endocytosis, and phagocytosis, thereby influencing cellular degradative processes and intracellular clearance [3,7,8].

Beyond its role in degradative pathways, TFEB also influences cell division and motility, metabolism, aging, immune responses, and vascular development, and it is involved in a various set of diseases, such as cancer, lysosomal storage diseases (LSDs), neurodegenerative diseases, metabolic disorders, and cardiovascular diseases [9,10,11,12,13,14,15]. The upregulation of catabolic processes mediated by TFEB allows tumorigenesis, and it is involved in the alterations of the tumor microenvironment [16,17,18,19].

Moreover, TFEB may play a role in angiogenesis, facilitating the formation of new blood vessels that supply oxygen and nutrients to the tumor and contributing to metastasis [17]. While the specific regulatory functions of TFEB in tumor angiogenesis remain under investigation, research conducted on embryos and newborn mice suggests that TFEB plays a role in the vascular development of endothelial cells (ECs). In particular, TFEB directly modulates the promoter region of the cyclin-dependent kinase 4 (CDK4) gene involved in cell cycle progression, and it indirectly influences the vascular endothelial growth factor receptor 2 (VEGFR2) gene expression through miR-15a/16-1 cluster regulation, suggesting the potential for miRNA-mediated regulation by TFEB [11].

MicroRNAs (miRNAs) are small non-coding RNAs, typically 19-25 nucleotides (nts) in length, playing a significant role in gene expression regulation. These miRNAs generally originate from the transcription of long primary miRNA transcripts, called pri-miRNAs, mediated by RNA polymerase II promoters. A specific pri-miRNA is subsequently processed in a ~100 nts hairpin-like intermediate known as pre-miRNA, which is further processed to release the mature -5p or -3p forms. Mature miRNAs bind to the 3’-untranslated region (3’-UTR) of target mRNAs, leading to the suppression of gene expression through either mRNA destabilization or translation inhibition [20].

A single mature human miRNA can potentially regulate various target genes. Conversely, a given human protein-coding transcript can be targeted by several miRNAs through the coupling of multiple binding sites in its 3′-UTR. Considering the comprehensive set of regulatory interactions among transcription factors, miRNAs, and the target genes expressed by the human genome, it is important to point out that the action of transcription factors and miRNAs in gene regulatory networks are not independent, but tightly intertwined. Such networks, particularly in human cells, likely consist of a complex array of mixed TF-miRNA regulatory circuits that modulate the functionality of each cell type [21,22].

In a previous study, our group employed a literature-mining approach to examine potential reciprocal interactions occurring between TFEB and miRNAs [23]. We found a potential sophisticated interplay between miRNAs, TFEB, and the regulated targets, highlighting how these types of crosstalk can affect TFEB activation and the corresponding cellular functions. However, to date, the comprehensive regulatory network orchestrated by TFEB remains poorly investigated, and no systematic molecular investigations are currently available for the dissection of TFEB–miRNAs relationships, in particular in endothelial models.

Building on this research, here, we present a multi-omics analysis aimed at exploring the role of TFEB’s regulatory network in the primary human umbilical vein ECs (HUVECs), serving as an endothelial model. By employing different high-throughput sequencing technologies, including chromatin immunoprecipitation (ChIP-seq), bulk transcriptome (mRNA-seq), and miRNA transcriptome (miRNA-seq) sequencing, we aimed to characterize the precise regulatory network driven by TFEB. Our findings indicate that TFEB regulates the expression of a broad spectrum of miRNAs across the human genome, potentially expanding its regulatory scope.

## 2. Results

### 2.1. Characterization of the TFEB Regulatory Landscape in HUVECs through ChIP-Seq

**Figure 1:**
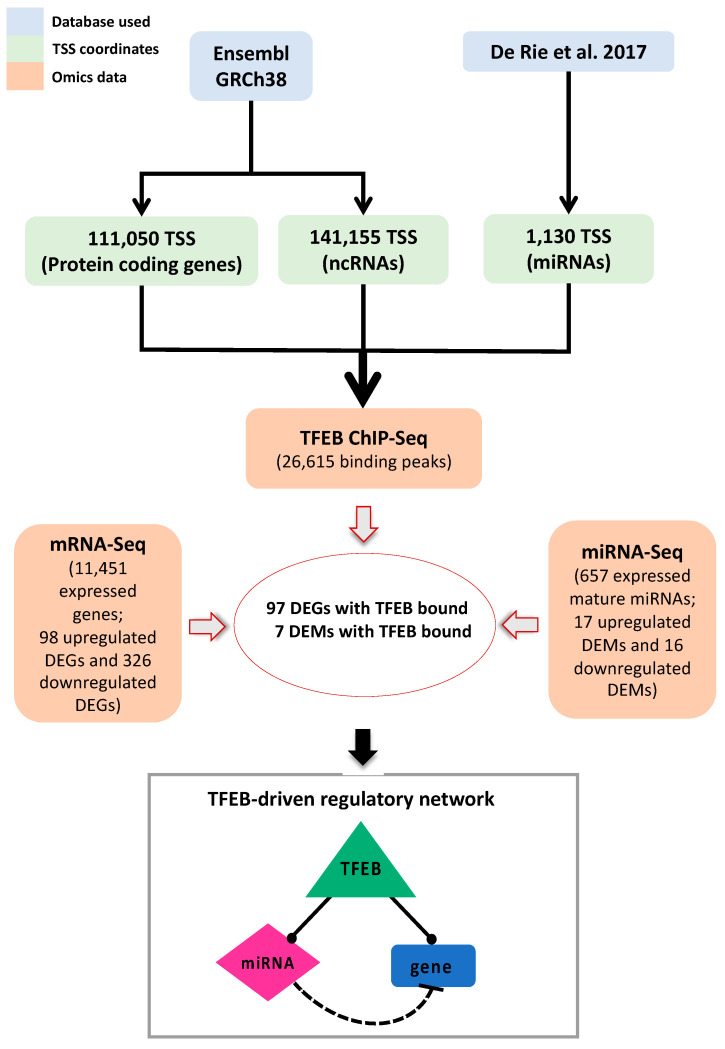
Overview of the bioinformatics analysis pipeline for defining the TFEB-driven regulatory network in the HUVECs. The blue rectangles represent the databases used to obtain annotations of transcription start sites (TSSs) on the human genome, depicted in green, for mRNAs, ncRNAs, and pri-miRNAs. The orange rectangles indicate the omics datasets used to identify the differentially expressed genes (DEGs) and the differentially expressed miRNAs (DEMs) showing a TFEB binding peak in their promoter regions. The integration of these data sets was conducted to identify a single comprehensive network of regulatory interactions, with TFEB as the master regulator (De Rie et al., 2017, [25]).

Figure 1 illustrates the use of an integrated bioinformatic approach employing high-throughput sequencing to elucidate the regulatory role of TFEB in primary HUVECs, specifically focusing on the role of miRNAs. To this end, we initially defined a comprehensive set of promoter regions associated with known transcripts in the human genome, encompassing both mRNAs and miRNAs. These promoter regions—associated with protein-coding mRNAs, non-coding-RNAs (ncRNAs), and miRNAs—were consistently identified to span from −2500 to +2500 bps around their respective transcription start sites (TSSs). The TSS coordinates for mRNAs and ncRNAs were retrieved from Ensembl [24], while those for miRNAs were derived from De Rie et al., 2017 [25].

We then assessed the binding of TFEB to these defined promoter regions using data from TFEB ChIP-seq in HUVECs. The raw data from TFEB ChIP-seq [11] were processed as described in the Section 4, revealing a total of 26,615 TFEB binding peaks with a −log10(p-value) > 3. The subsequent analysis of the intersection between these TFEB peaks and the predicted promoter regions yielded a total of 5588 protein-coding mRNAs and 298 mature miRNAs that showed at least one TFEB binding peak in their promoter regions (Supplementary Table S1).

Having obtained the list of mRNAs and miRNAs potentially regulated by TFEB through direct promoter binding, we further investigated the TFEB-driven regulatory network in the HUVECs by integrating mRNA-seq and miRNA-seq data to identify differentially expressed genes (DEGs) and differentially expressed miRNAs (DEMs) upon TFEB silencing. This integration allowed us to characterize the final TFEB-driven regulatory network, which is schematically depicted in Figure 1 and discussed in detail in paragraph 2.4. This network illustrates the complex interactions among these molecules and underscores the broad regulatory influence exerted by TFEB in EC functions.

### 2.2. mRNA-Seq Reveals Differential Gene Expression Patterns Associated with TFEB in HUVECs

**Figure 2:**
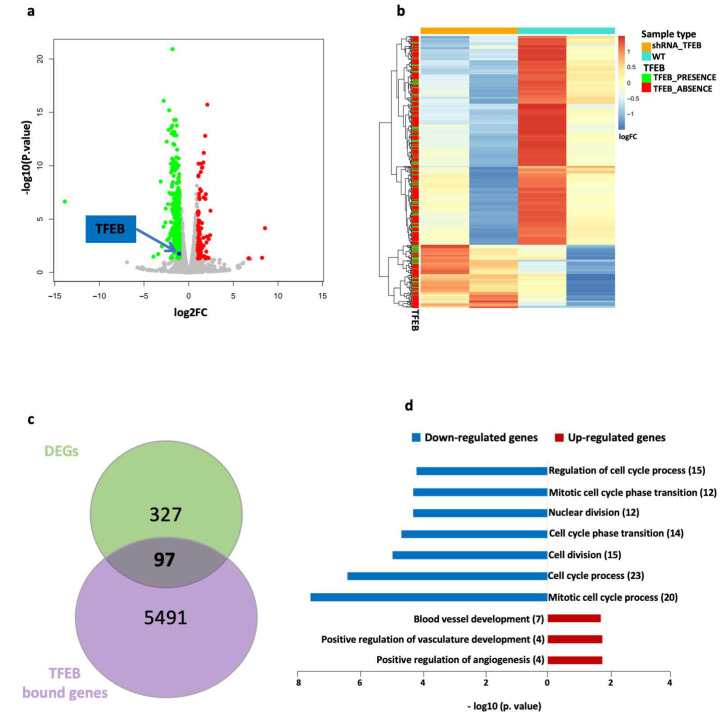
mRNA-Seq data analysis reveals a TFEB role in regulating protein-coding and ncRNA genes in HUVECs. (**a**) Volcano plot showing differentially expressed genes (DEGs) in TFEB-silenced (shRNA-TFEB) HUVECs compared to control cells (WT). The green dots represent the downregulated genes, while the red dots represent the upregulated ones. The blue dot indicates the TFEB gene itself. (**b**) Heatmap showing unsupervised hierarchical clustering of DEGs between the TFEB-silenced HUVECs (orange) and WT (cyan). Each DEG is annotated to show the presence (green) or absence (red) of a significant TFEB binding event in its promoter region. (**c**) Venn diagram depicting the overlap between DEGs in TFEB-silenced HUVECs and WT cells, along with the total number of genes bound by TFEB in their promoter regions. (**d**) Selected GO terms obtained from the ToppGene functional analysis of the DEGs. The GO terms associated with downregulated genes are shown in blue, while those associated with upregulated genes are red. The number of genes associated with each term is indicated in parentheses. GO terms are ranked by –log10(p-value), adjusted using the Benjamini–Hochberg method.

To elucidate the genetic program modulated by TFEB in primary HUVECs, we assessed the transcriptome alterations occurring upon TFEB silencing and correlated them with the DNA promoter regions where TFEB was recruited. For this purpose, we conducted a bulk mRNA-seq experiment comparing two different biological conditions: one in which TFEB was down-modulated by a specific sh-RNA, and another representing control HUVECs infected with a non-target sh-RNA. Following gene quantification, only genes with an expression pattern of transcripts per million (TPM) > 1 in at least one replicate were considered expressed, effectively reducing background noise. A total of 11,451 expressed genes were identified. Among these, 424 genes were DEGs between the TFEB-silenced HUVECs and the control HUVECs (Supplementary Table S2, Supplementary Figure S1). These modulated genes are depicted in the volcano plot in Figure 2a, with 98 upregulated (shown in red), and 326 downregulated genes (shown in green).

Upon intersecting these genes with the TFEB ChIP-seq data, we observed that out of the 424 DEGs from TFEB-silenced cells, 97 (25 upregulated and 72 downregulated) exhibited a TFEB binding peak in their promoter region, while 327 did not. A heatmap depicting the unsupervised hierarchical clustering of these modulated genes, along with the information about TFEB binding sites in their promoter regions, is presented in Figure 2b. Figure 2c shows a Venn diagram summarizing the number of genes bound by TFEB and the number of DEGs upon TFEB silencing.

To investigate the biological role of these modulated genes regulated by TFEB, we performed a functional analysis via ToppGene [26], particularly focusing on biological functions. We observed that several gene ontology (GO) terms related to cell cycle and mitosis regulation were predominant among the downregulated genes, whereas the GO terms associated with angiogenesis and vascular development were more prevalent among the upregulated ones (Figure 2d).

### 2.3. TFEB Transcriptional Regulation of miRNAs in HUVECs

**Figure 3:**
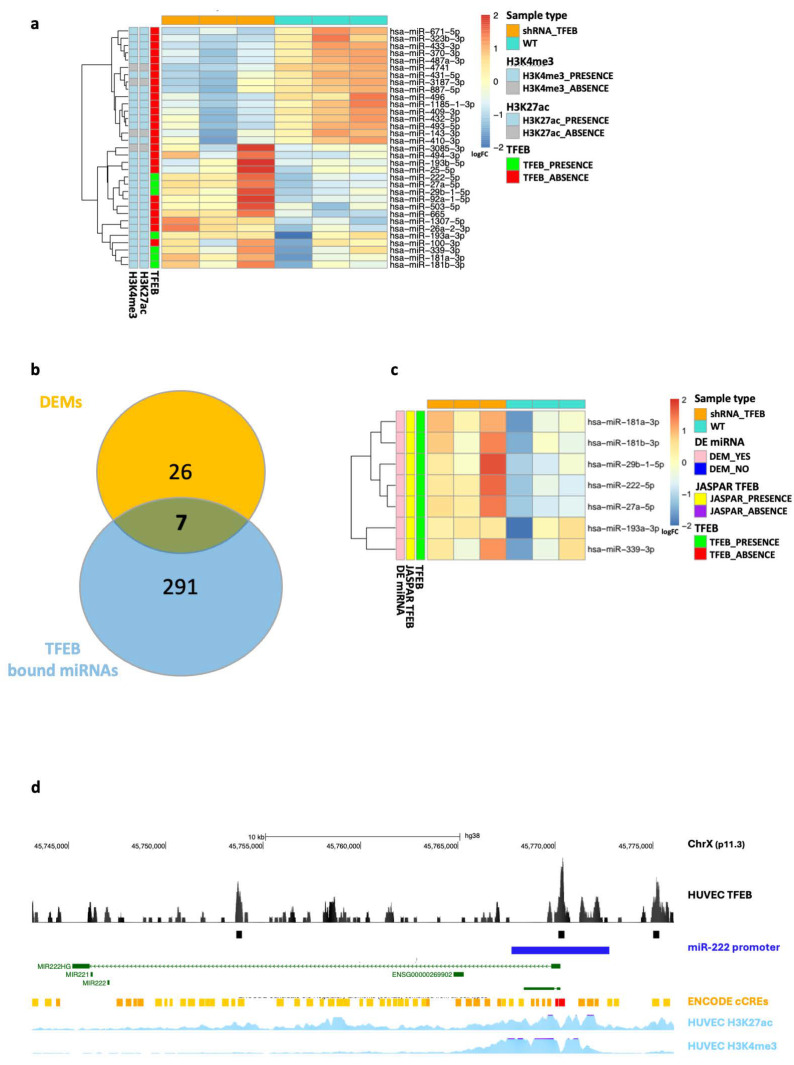
TFEB-mediated regulation of miRNAs in HUVECs. (**a**) Heatmap showing the unsupervised hierarchical clustering of differentially expressed miRNAs (DEMs) between the TFEB-silenced (shRNA-TFEB) (orange) and control (WT) HUVECs (cyan). Each miRNA is annotated to indicate the presence (green) or absence (red) of a TFEB significant binding event in its promoter region. In addition, each miRNA is annotated to show whether the H3K27ac and H3K4me3 marks are present (light blue) or absent (light grey) in its associated promoter region, according to ENCODE data from HUVECs. (**b**) Venn diagram showing the overlap between miRNAs among TFEB-silenced HUVECs and WT groups and the total number of miRNAs bound by TFEB in their promoter regions. (**c**) Heatmap showing the unsupervised hierarchical clustering of differentially expressed miRNAs (annotated in pink) characterized by TFEB in their promoter region (annotated in green) between the TFEB-silenced HUVECs (orange) and WT conditions (cyan). Each miRNA is also annotated to display the presence (yellow) or absence (purple) of the TFEB motif in its associated promoter region, based on data from the JASPAR database. (**d**) Graphical representation of the miRNA-222 locus on the human genome hg38, sourced from the UCSC Genome Browser. The graphic includes annotations for the miR-222 host gene (green) and its putative promoter region (−2500, +2500 DNA bases around the TSS, blue). At the top, the TFEB binding signal in HUVECs is shown in black. At the bottom, the data for H3K27ac and H3K4me3 from ENCODE HUVECs are shown.

In parallel with our mRNA-seq analysis, we also conducted miRNA expression analysis using miRNA-seq in the same biological model of primary TFEB-silenced HUVECs. A total of 657 mature miRNAs were detected, out of which 33 were differentially expressed—17 upregulated and 16 downregulated—upon TFEB silencing compared to the control (Supplementary Table S3). Of note, among these 33 DEMs, seven showed a TFEB binding event in their promoter region, while 26 did not. Further analysis revealed that 29 of the 33 miRNAs showed H3K27ac and H3K4me3 signals in their associated promoter regions, indicating active transcriptional regions according to the HUVECs ENCODE data [27,28], as represented in the heatmap in Figure 3a and the Venn diagram in Figure 3b.

All seven of the miRNAs modulated upon TFEB silencing and associated with a TFEB binding event in their putative promoter regions were upregulated. To further confirm the presence of the binding motif of TFEB in their promoter region, we analyzed the promoter sequence using data from the JASPAR database [29]. Figure 3c shows the heatmap representing the unsupervised hierarchical clustering of the expression data of these seven selected DEMs (i.e., hsa-miR-181a-3p, hsa-miR-181b-3p, hsa-miR-29b-1-5p, hsa-miR-222-5p, hsa-miR-27a-5p, hsa-miR-193a-3p, and hsa-miR-339-3p), along with the annotation of the TFEB motif in their promoter regions.

Given the biological significance of these findings, we chose to focus our attention on miR-222, which is known to negatively regulate the angiogenic activity of the stem cell factor (SCF) by targeting its receptor, c-Kit [30]. Figure 3d illustrates the genomic region encompassing the miR-222 locus on the reference human hg38 genome, as depicted by the UCSC Genome Browser [31]. This region not only shows a TFEB binding peak in the promoter region of the pri-miRNA but also displays additional signals from the H3K27ac and H3K4me3 HUVEC tracks obtained from ENCODE, supporting the notion of an actively transcribed region and highlighting the complex regulation by TFEB in the EC environment.

### 2.4. TFEB Mediates miRNA Regulatory Network in HUVECs

**Figure 4:**
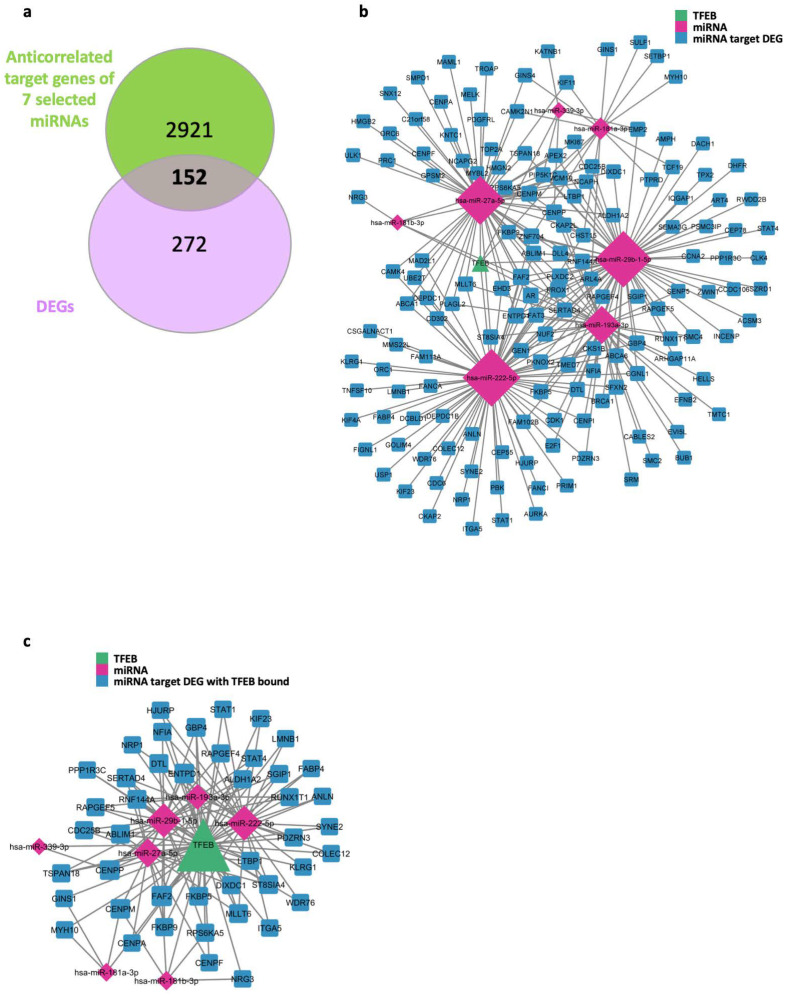
TFEB/miRNAs-mediated regulatory networks (**a**) Venn diagram representing the overlap between the number of anti-correlated target genes of the 7 DEMs between TFEB-silenced (shRNA-TFEB) and control (WT) HUVECs, with respect to the DEGs identified when comparing TFEB-silenced to WT HUVECs. These target genes are further characterized by the presence of TFEB binding sites in their promoter regions. (**b**) TFEB-mediated network featuring the 7 DEMs with TFEB presence in their promoter regions, along with their 152 differentially expressed target genes. Specifically, TFEB is indicated by green triangles, miRNAs by magenta diamonds, and differentially expressed target genes by blue squares. Notably, some of these target genes exhibit TFEB binding in their promoter regions, while others do not. (**c**) A focused subset of the broader regulatory network that specifically includes TFEB, the 7 DEMs, and their 44 differentially expressed target genes, where each node is marked by the presence of TFEB binding in its associated promoter region. This subset underscores the specific interactions within the network, where TFEB more likely directly influences gene expression through its binding activity.

Focusing on the subset of seven DEMs showing a TFEB binding peak in their promoter regions, we sought to determine their roles in gene regulation. For this purpose, we determined their target genes using the TargetScan Human database [32]. Specifically, we considered those target genes that were modulated in the RNA-Seq data upon TFEB silencing and exhibited anti-correlation with respect to the miRNA expression data. Our analysis detected 3,073 anti-correlated target genes associated with these seven DEMs. Noteworthy, 152 of these were also DEGs between the TFEB-silenced HUVECs and the control (Figure 4a).

Figure 4b outlines the regulatory network formed by the seven DEMs, each characterized by a TFEB binding event at their promoter regions, and their corresponding set of 152 differentially expressed target genes upon TFEB silencing.

Of the 152 differentially expressed genes, 44 showed TFEB binding in their promoter regions, while 108 did not. To further characterize this landscape, Figure 4c displays a TFEB-mediated miRNA–target gene interaction network, focusing specifically on those target genes bound by TFEB (Supplementary Table S4).

### 2.5. TFEB Modulates E2F1 and CDK1 via miR-222 in HUVECs

**Figure 5:**
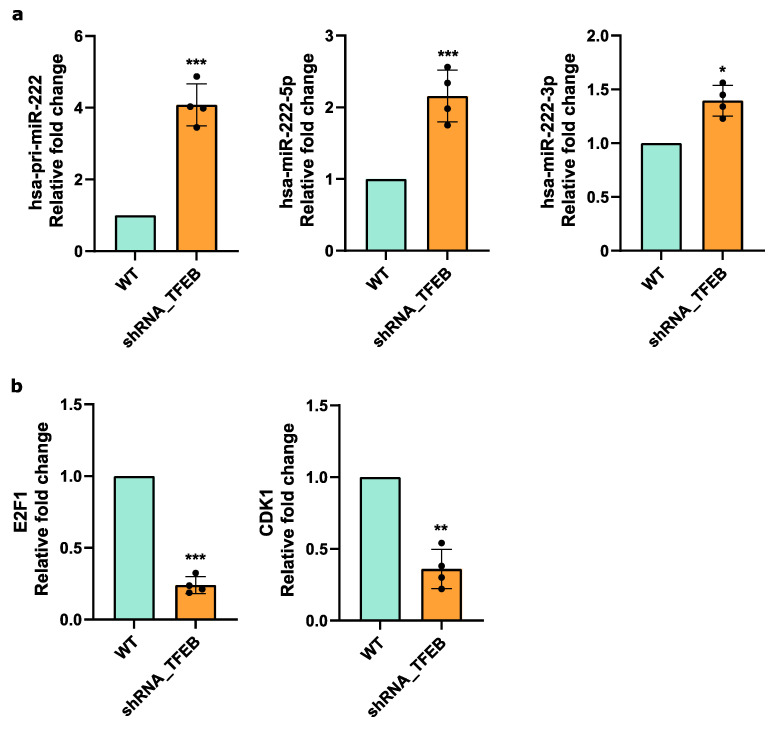
TFEB-miR-222-E2F1/CDK1 network in HUVECs (**a**,**b**): quantitative PCR (qPCR) analysis of hsa-pri-miR-222 and mature hsa-miR-222-5p or 3p (**a**), alongside E2F1 and CDK1 (**b**), in TFEB-silenced (shRNA-TFEB) or WT HUVECs. Data are expressed as relative fold-change compared to the expression in WT cells, normalized to the housekeeping gene TBP (n = 4, mean ± SEM; * *p*-value < 0.01, ** *p*-value < 0.001 and *** *p*-value < 0.0001, determined by Student’s *t*-test).

MiRNA-seq analysis demonstrated that the miR-222 expression levels were downregulated by TFEB (Figure 3a–d). Furthermore, bioinformatics analysis based on the TFEB ChIP-Seq binding dataset detected an enrichment of TFEB at the miR-222 promoter region, suggesting a direct regulatory influence of the transcription factor on miR-222 (Figure 3d). To confirm this assumption, we assessed the expression levels of both pri-miR and mature miR-222-3p/5p in the control and TFEB-silenced HUVECs. As expected, TFEB silencing led to an increase in both the pri-miR and miRNA expression levels (Figure 5a).

Anti-correlation analysis identified several miR-222 target DEGs, among which were the transcription factor E2F1 and Cyclin Dependent Kinase 1 (CDK1), known for their role in regulating cell cycle progression [33]. Consistent with the upregulation of miR-222 following TFEB silencing, E2F1 and CDK1 expression was downregulated in the HUVECs (Figure 5b), thereby confirming our predictive data.

Taken together, these findings underscore the role of TFEB in modulating key cell cycle regulators indirectly through miRNA-mediated pathways in ECs.

## 3. Discussion

TFEB acts as a transcriptional regulator with crucial roles in autophagic, lysosomal, and endothelial processes. It also significantly contributes to endothelial functions and stability, which are important for maintaining vascular integrity.

In this study, we aimed to further characterize the molecular mechanisms underlying TFEB’s transcriptional activity within the endothelial regulatory network, using primary HUVECs as a reliable cell model [34]. To this end, we have established a bioinformatic pipeline to integrate different high-throughput sequencing data, including ChIP-seq, mRNA-seq, and miRNA-seq in HUVECs. More specifically, the mRNA-seq and miRNA-seq experiments were conducted in a model of TFEB-silenced HUVECs, enabling us to define the regulatory network of TFEB, with a particular focus on the role of miRNAs in mediating TFEB’s transcriptional activity. A key accomplishment of our work is, therefore, the creation a comprehensive high-throughput dataset of gene and miRNA expression in a biological model that accurately represents the endothelium, directly linking it to TFEB modulation (Supplementary Tables S5 and S6).

The human regulatory network is defined by complex, reciprocal interactions among transcription factors, miRNAs, and target genes, with miRNA-mediated regulatory circuits representing a key feature of these sophisticated systems. Here, we provide a comprehensive set of experimental data, supported by unbiased bioinformatic analysis, demonstrating the extensive involvement of miRNA-mediated regulatory circuits in modulating the activity of TFEB in ECs (Figure 2, Figure 3 and Figure 4). The significant role of non-coding RNAs, particularly miRNAs, as identified in our study, is corroborated by several other works characterizing endothelial models, even in significantly different contexts [35,36,37].

Our analysis began by examining the overlap between TFEB ChIP-seq data in HUVECs and a set of putative promoter regions on the human genome, covering both mRNA genes and pri-miRNAs. We observed a significant presence of TFEB binding peaks in the promoter regions of both DEGs and DEMs upon TFEB silencing, suggesting a direct involvement of TFEB in their transcriptional regulation. However, given that the collection of promoter regions from the TSS catalog we employed is likely incomplete, it is tempting to speculate that more updated versions may reveal additional regulatory interactions mediated by TFEB [38]. This appears to be particularly relevant for miRNAs that are known to have alternative promoters other than the regions surrounding the TSS of the pri-miRNAs used in this study [39]. Moreover, just a small portion of the total number of TFEB binding peaks overlapped with known ENCODE candidate cis-regulatory elements (cCREs) of a promoter-like type in the human genome, whilst the vast majority of them were associated with distal enhancer elements (Supplementary Figure S2). This observation suggests the existence of an even broader regulatory network sustaining ECs upon TFEB silencing, where distal or indirect interactions play a central role. Finally, it is important to note that the interaction pattern between TFEB and miRNA proposed in this study (Figure 1 and Figure 4) represents just one of a series of potential miRNA-mediated regulatory circuits that could operate in a living cell [21]. Additional regulatory circuits could be reconstructed from analyses not solely based on the use of anti-correlation patterns between expression data, as demonstrated here. Experiments are ongoing to further address these important aspects.

Interestingly, a portion of the genes and miRNA identified in our analyses appear to be negatively regulated by TFEB. We have already explored TFEB’s potential role as a negative regulator and demonstrated its direct negative regulation in a specific example, where TFEB negatively regulates MYO1C in HUVECs [11]. However, the presence of negatively regulated targets could also stem from additional indirect regulations or the presence of specific transcriptional cofactors.

In the concluding phase of our study, we focused on seven DEMs characterized by TFEB binding peaks in their promoter regions upon TFEB silencing: miR-181a-3p, miR-181b-3p, miR-29b-1-5p, miR-222-5p, miR-27a-5p, miR-193a-3p, and miR-339-3p. These miRNAs are integral to various ECs functions and vascular biology, including vascular inflammation, angiogenesis, endothelial integrity, and vascular remodeling [40,41,42,43,44,45,46,47,48]. Below, we briefly summarize the specific impact of each miRNA on vascular health and their related references:

– miR-181a-3p is associated with slowed progression of atherosclerosis due to its role in inhibiting vascular inflammation. Specifically, it exerts an anti-inflammatory effect in ECs by reducing the expression of adhesion molecules, such as intercellular adhesion molecule 1 (ICAM-1) and vascular cell adhesion protein 1 (VCAM-1), as well as NF-κB essential modulator (NEMO), which is involved in NF-κB signaling [40];
– miR-339-3p is upregulated in vascular smooth muscle cells (VSMCs) in response to angiotensin II receptor-1 autoantibody (AT1-AA), contributing to vascular inflammation [41];
– miR-181b-3b is overexpressed in chronic obstructive pulmonary disease (CODP), where it is associated with pulmonary endothelial dysfunction. Its overexpression in HUVECs in vitro leads to reduced endothelial sprouting and, consequently, diminished angiogenesis [42];
– miR-29b-1-5p is downregulated in damaged endometrial stromal cells (ESCs) co-cultured with Wharton’s jelly mesenchymal stem cells (WJ-MSCs), which, through the upregulation of RAPB1, positively influences angiogenesis [43];
– miR-222-5p is known for negatively regulating the angiogenic activity of the stem cell factor (SCF) by targeting c-Kit [30]. It is also upregulated in the vascular smooth muscle cells (VSMCs) treated with oxidized low-density lipoprotein (ox-LDL), contributing to the development of atherosclerosis [44];
– miR-27a-5p is downregulated in exosomes derived from Tsp-1-expressing microglia, leading to increased Smad3 expression in ECs, which reduces retinal neovascularization, suggesting a protective role in maintaining vascular homeostasis and suppressing pathological angiogenesis [45];
– miR-193a-3p is involved in the pathogenic mechanisms of vascular conditions, such as obstructive sleep apnea (OSA), aortic dissection (AD), and angiogenesis. It regulates endothelial functions in HUVECs, VSMCs, and in circulating endothelial colony-forming cells (ECFCs) [46,47,48].

Among these seven miRNAs, we decided to validate miR-222 and its targets, DEGs E2F1 and CDK1, as they are involved in one of the most specific functions of TFEB in the endothelium [11], i.e., the regulation of the progression of the cell cycle from the G1 phase to the S phase. In line with our prediction, the silencing of TFEB led to increased expression of both pri-miR and mature miR-222, accompanied by E2F1 and CDK1 downregulation, indicating a direct regulatory interaction between TFEB and miR-222 in HUVECs, which constitutes a novel paradigm of TFEB-mediated EC regulation (Figure 5).

The network depicted in Figure 4a is composed of miRNAs and genes that are a subset of those identified as differentially expressed following TFEB silencing. To investigate the biological significance of the subset of genes reported in Figure 4a, we utilized ToppGene again to identify enriched gene ontology functions. The analysis confirms enrichment for functional categories related to replication and the cell cycle 

To our knowledge, apart from HUVECs, TFEB ChIP-Seq analyses have been conducted in HeLa, human EndoC-βH1, and HEK293 cell lines [4,6,49,50]. However, in none of these has the ability of TFEB to regulate miRNAs been analyzed in detail. Therefore, our study represents the first comprehensive catalog of a TFEB-driven miRNA-mediated regulatory network in the HUVECs and introduces a novel perspective by revealing, for the first time, the crucial role of TFEB in orchestrating regulatory activities mediated by non-coding RNAs.

## 4. Materials and Methods

### 4.1. Cells and Genetic Manipulation

In vitro experiments were performed on primary HUVECs isolated from umbilical cord veins maintained as described previously [11] and used between passage 1 and 3. HUVECs from a pool of five different donors were used in all experiments to minimize cell variability [51]. The presence of mycoplasma contamination was routinely checked using a Venor GeM Mycoplasma Detection kit (ThermoFisher Scientific, Waltham, MA, USA).

Endogenous *TFEB* was silenced in HUVECs using a specific sh-RNA lentivirus. In all experiments, TFEB-silenced cells (shRNA-TFEB) were compared to control cells transduced with a scramble shRNA (WT). The experiments were performed using different sh-RNA-TFEB constructs, all of which demonstrated comparable efficiency and specificity, as previously described [11]. In particular, for the entire study, we employed shRNA-TFEB TRCN000001311, which was cloned in a pLKO.1-puro nonmammalian vector. HUVECs were transduced with these specific lentiviral particles at a multiplicity of infection (MOI) of 1, prepared according to Follenzi et al. [51]. After 24 h, the medium was replaced, and cells stably expressing the lentivirus were selected on puromycin (1 μg/mL) for 24 h. As previously described [11], the inhibition of TFEB in HUVECs was confirmed by RNA-seq analysis (Figure 2a) and by qPCR (Supplementary Figure S3).

### 4.2. mRNA-Seq Sample Preparation

Approximately 200 ng of high-quality RNA (RIN > 9) was utilized to prepare RNA-libraries using TruSeq Stranded Total RNA Kit (Illumina, San Diego, CA, USA) according to the manufacturer’s instructions. Library quality was checked with a Bioanalyzer DNA High Sensitivity assay (Agilent, Santa Clara, CA, USA). Libraries were then multiplexed, clustered, and sequenced for poly-A^+^ RNAs on an Illumina NovaSeq 6000, using a paired-end protocol, at an external next generation sequencing (NGS) facility (Fasteris, Geneva, Switzerland). The experiment was performed with two biological replicates to ensure data reliability.

### 4.3. miRNA-Seq Sample Preparation

Small-RNA-sequencing was performed according to the Illumina TruSeq small RNA protocol (Illumina, San Diego, CA, USA), as per manufacturer’s instructions. The first step of the Standard pipeline suggested an acrylamide gel size selection to purify small RNA, followed by library construction using Illumina TruSeq small RNA kit. High-quality RNA (RIN > 9) was subjected to acrylamide gel size selection to purify small RNA before library preparation and to improve the quality of the library. The selected small RNAs typically ranged from 18 to 30 nt. Library quality was verified through a Bioanalyzer DNA High Sensitivity assay (Agilent, Santa Clara, CA, USA). Libraries were then multiplexed, clustered, and sequenced on an Illumina NovaSeq 6000, using a single-end protocol, at an external NGS facility (Fasteris, Geneva, Switzerland). To ensure the robustness and reproducibility of the data, all experiments were performed with three biological replicates.

### 4.4. mRNA-Seq Analysis

The Fastq files generated were initially assessed for quality using FastQC software [52]. Subsequently, TrusSeq adapters were trimmed using Cutadapt v3.5 [53] in paired-end mode. After this step of reads trimming, a set of good quality Fastq files was obtained. At this point, RSEM v.1.3.1 [54] pipeline was launched using STAR aligner [55] in paired-end mode to quantify genes expressed in all samples using human hg38 assembly as the reference genome. Annotations of genes provided by the Ensembl database [24] were set as reference for the quantification of gene expression levels. A list of DEGs between TFEB-silenced (shRNA-TFEB) and control HUVECs (WT) was subsequently obtained using DESeq2 [56] with |log2FC| > 1 and FDR < 0.05 as statistical significance thresholds.

### 4.5. miRNA-Seq Analysis

The good quality of reads was assessed using FastQC software [52]. The analysis of Fastq files proceeded through a miRDeep2 v. 2.0.1.2-based pipeline [57], applying default parameters to both the mapper.pl and miRDeep2.pl scripts; hg38 served as the reference. The database for genomic annotation of miRNAs and for linking miRNA mature IDs with pre-miRNA and pri-miRNA IDs was mirBase, version 22.1 [58]. The output from the miRDeep2 pipeline was then filtered to obtain a list of expressed miRNAs. This consisted in calculating the average value of expression per each pair of mature miRNAs among the precursor miRNAs, retaining a final table of mature miRNAs that had a normalized count > 1 in at least one sample. Subsequently, the DEMs between TFEB-silenced (shRNA-TFEB) and control HUVECs (WT) were determined using DESeq2 [56] applied to miRNA raw counts. The criteria set for identifying significant differential expression were |log2FC| > 0.5 and a false discovery rate (FDR) < 0.1.

### 4.6. ChIP-Seq Analysis

The good quality of reads was assessed using FastQC software [52]. Publicly available Fastq files from ChIP-seq of HUVECs overexpressing TFEB and the corresponding input file were obtained from Doronzo et al. [11]. These files were downloaded following the links in NCBI under accession GEO GSE88896 and the specific SRA links SRR4429371 (ChIP-seq TFEB) and SRR4429370 (Input_igG) using fastq-dump (https://trace.ncbi.nlm.nih.gov/Traces/sra/sra.cgi?view=software, version 3.0.6, accessed on 1 May 2024).

ChIP-Seq data were analyzed using Bowtie2 [59] as the aligner, applying default parameters with hg38 as the genome reference. Subsequently, Macs2 (v2.1.1.20160309) callpeak function [60] was used to call the binding region of TFEB across the genome. The parameters set for comparing the TFEB HUVEC ChIP sample to the corresponding input file included: -f BAM -g hs -n output_name -B -p 0.01 --call-summits --fe-cutoff 1.0.

### 4.7. Bioinformatic Data Integration

The promoter regions for protein-coding and non-coding-RNA (ncRNA) genes were defined by downloading a custom list from the Ensembl BioMart database for the hg38 assembly, v100 [24]. These regions spanned from −2500 to +2500 bps around the TSSs of each Ensembl transcript, resulting in a dataset of 252,205 putative promoters. This list was then intersected with macs2 output peaks using BEDTools [61], with the “-wao” option to annotate peaks overlapping these promoter regions. Multiple TFEB hits for each gene were merged and further refined to include only those with a −log10(p-value) > 3 and an overlap > 100 nts as thresholds, establishing a final list of genes associated with TFEB-bound promoters.

A list of TSSs for human pri-miRNAs was initially sourced from De Rie et al. 2017 [25]. We then identified a set of putative promoter regions for each human miRNA by defining a region spanning from −2500 to +2500 base pairs (bps) around each of the available human miRNA TSS, resulting in a set of 1,130 putative pri-miRNA promoters. As the coordinate system used by De Rie et al. 2017 was based on the hg19 reference genome, we converted these coordinates to align with the hg38 reference genome using the LiftOver tool from the UCSC Genome Browser [31]. TFEB ChIP-Seq peaks, with scores indicating a −log10(p-value) > 3, were intersected with this list of miRNA promoter regions in hg38 coordinates using the BEDTools intersect command, with the “-wao” option [61]; only peaks showing an overlap > 100 nts were considered as a positive overlap and retained for further analysis.

H3K27ac and H3K4me3 hg38 peaks in HUVECs were accessed from the ENCODE portal [27,28] (https://www.encodeproject.org/, accessed on 1 May 2024) using the following identifiers: ENCFF426QFH for the H3K27ac and ENCFF992YLK for H3K4me3 peaks.

The DEMs that had TFEB binding around their promoter regions were further annotated for the presence of TFEB motifs in their promoter regions using the MA0692.1 matrix profile from the JASPAR database (9th release) [29].

The target genes of DEMs were identified using TargetScan Human version 8.0 [32]. An anti-correlation analysis was then applied, retaining only those genes which were differentially expressed and showed an anti-correlated expression pattern with respect to the regulating miRNAs. Specifically, we selected pairs of DEMS and DEGs with [log2FC_miRNA > 0 and log2FC_gene < 0] or [log2FC_miRNA < 0 and log2FC_gene > 0], respectively. From this analysis, we defined two distinct networks: the first includes edges connecting TFEB, DEMs, and DEGs regardless of TFEB binding in their promoter regions; the second one consists of connections between TFEB, DEMs, and DEGs with TFEB binding in their promoter regions.

The networks illustrating the anti-correlated regulatory relationships between TFEB, miRNA, and genes were visually represented using Cytoscape, version 3.9.1 [62].

Volcano plots were computed using the scatter plot function in R, and heatmaps were generated using the “pheatmap” package in R. Finally, graphical representations of selected miRNA promoter regions were obtained using the UCSC Genome Browser [31].

### 4.8. Functional Annotation of Differentially Expressed Genes

The list of downregulated and upregulated genes obtained by the mRNA-seq analysis was used as input for a functional analysis with ToppGene [26]. We focused on selected gene ontology (GO) biological process terms, which were ranked based on their statistical significance, determined through p-values corrected using the Benjamini–Hochberg method.

### 4.9. Real-Time PCR

After extraction with Maxwell^®^ RSC miRNA tissue kit (Promega Corporation, Madison, WI, USA), RNA concentration and quality were checked on a NanoDrop 2000 spectrophotometer (Thermo Fisher Scientific, Waltham, MA, USA) and on a Bioanalyzer 2000 (Agilent, Santa Clara, CA, USA), respectively.

RNA was converted to cDNA using a High-Capacity cDNA Reverse Transcription kit. To analyze miRNA expression levels, RNA was reverse-transcribed using a High-Capacity cDNA Reverse Transcription kit in the presence of miRNA-specific primers (ThermoFisher Scientific, Waltham, MA, USA). Real-time PCR was performed on a CFX96 system (Bio-Rad) using TaqMan Universal PCR Master Mix (ThermoFisher Scientific, Waltham, MA, USA) or miRNA-specific TaqMan MicroRNA assays using gene- or miRNA-specific TaqMan assays. TBP and RNU44 were used as reference genes. The following Taqman assays (ThermoFisher Scientific, Waltham, MA, USA) were used: TFEB (human, Hs00292981_m1), E2F1 (human Hs00153451_m1), and CDK1 (human Hs00938777_m1); pri-miR-222 (Hs03303011_pri); miR-222-3p (002276); miR-222-5p (002097); TBP (human Hs00427620) and RNU44 (001094).

### 4.10. Statistical Analysis

The sample sizes were not determined through a specific power analysis but were chosen based on sizes commonly used in similar studies in the field of vascular development, as referenced in the specific literature. Thus, no statistical methods were used to predetermine the sample size. Furthermore, we did not randomize the samples because our experimental design did not require this type of strategy. Moreover, the investigators were not blinded to the allocation of the samples during the experiments and analyses.

The data are reported as the mean ± SEM. All statistical analyses were performed using Excel (Microsoft, v16.86) and Prism (GraphPad, v8.0.1) software. Appropriate statistical tests were performed as indicated in the Section 2. A p-value < 0.05 was considered statistically significant for all the experiments.

## 5. Conclusions

In conclusion, our findings indicate the important role of TFEB in regulating a potentially extensive network of miRNAs in the endothelium. We have uncovered a dynamic interplay between miRNAs and endothelial gene expression, both regulated by TFEB, identifying specific miRNA-mediated regulatory circuits that position TFEB as a novel master regulator in ECs. Our findings are further supported by experimental validations, specifically involving miR-222, confirming the reliability of our results.

Overall, our findings highlight the need for additional experiments to further clarify the roles of the individual components of the proposed TFEB-driven regulatory network and their implications in vascular biology and disease therapeutics.

## Data Availability

TFEB ChIP-seq data in HUVECs were originally published by some of us in [11] and are available in the Gene Expression Omnibus of the National Center for Biotechnology Information (accession No. GSE88896). mRNA-seq and microRNA-seq data upon TFEB silencing in HUVECs were instead produced for this study. Requests for raw sequencing data access can be addressed to the corresponding authors.

## Supplementary Materials

The following supporting information can be downloaded at: https://www.mdpi.com/article/10.3390/ijms25137123/s1.
