# Supplementary material for: Integrative Bioinformatics Analysis Reveals a Transcription Factor EB-Driven MicroRNA Regulatory Network in Endothelial Cells
Source: Int J Mol Sci. 2024 Jun 28;25(13):7123. doi: 10.3390/ijms25137123 (PMC11241138; doi:10.3390/ijms25137123)
Supplement: Supplementary file 1 [file ijms-25-07123-s001.zip › Supplementary_Figures.pdf]

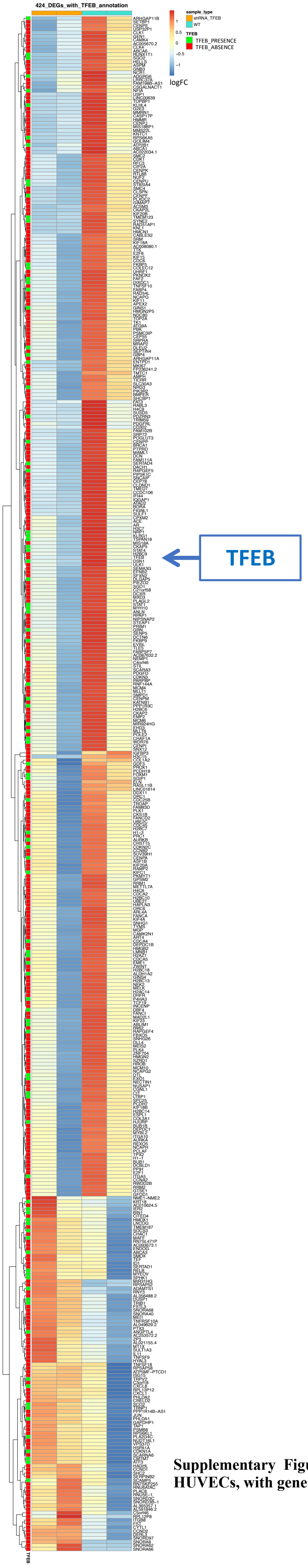

Supplementary Figure S1. Heatmap of the 424 genes DEGs between TFEB-silenced HUVECs and control HUVECs, with gene names highlighted.

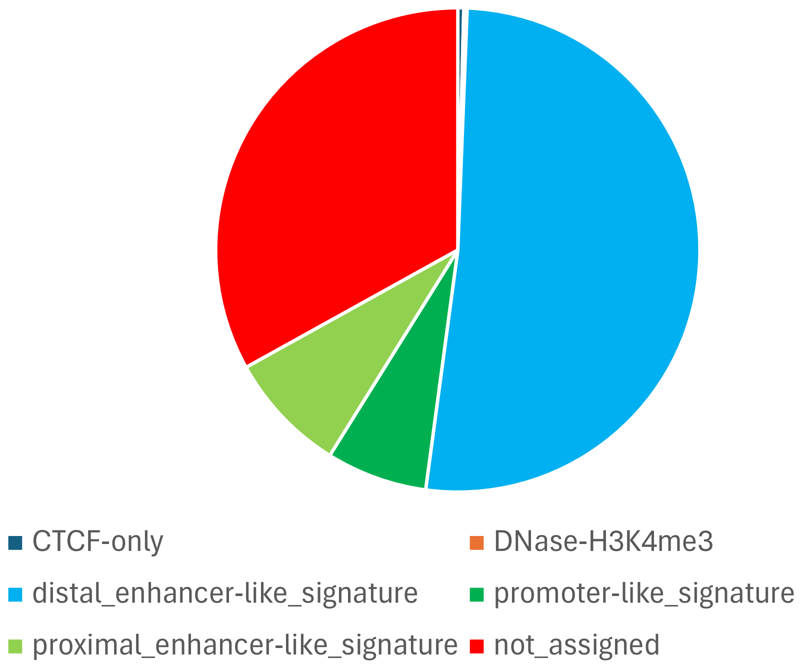

ENCODE cCRE type	% of HUVEC TFEB overlapping peaks
CTCF-only	< 1.0
DNase-H3K4me3	< 1.0
distal_enhancer-like_signature	51.0
promoter-like_signature	7.0
proximal_enhancer-like_signature	8.0
not_assigned	33.0

Supplementary Figure S2. Overlap between TFEB HUVEC peaks and ENCODE cCREs. Pie-chart showing the overlap between the catalog of TFEB HUVEC peaks identified in HUVECs and candidate cis-regulatory elements (cCREs) in the human genome annotated by the ENCODE project. The accompanying details the percentages of overlaps between TFEB peaks and each class of cCREs, considering only cases with overlaps > 100 nts.

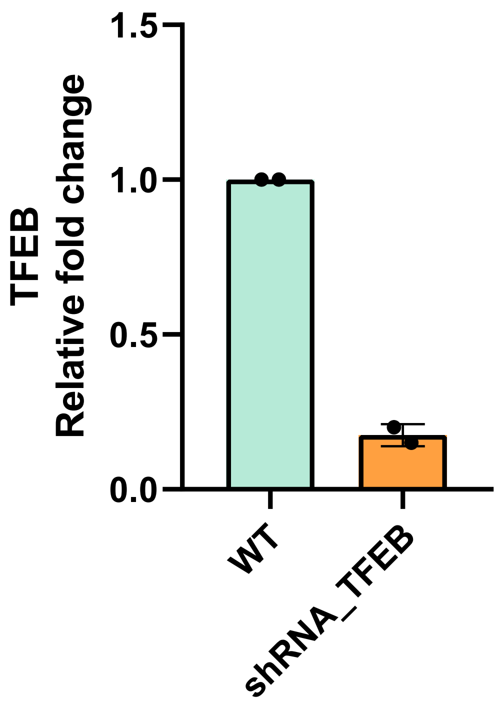

Supplementary Figure S3. Quantitative PCR (qPCR) analysis of TFeb in TFEB-silenced (shRNA-TFEB) or WT HUVECs. Data are expressed as relative fold-change compared to the expression in WT cells, normalized to the housekeeping gene TBP (n = 2, mean ± SEM; ****p* < 0.0001, determined by Student's t-test).
